# Artificial Intelligence (AI) Assisted CT/MRI Image Fusion Technique in Preoperative Evaluation of a Pelvic Bone Osteosarcoma

**DOI:** 10.3389/fonc.2020.01209

**Published:** 2020-08-04

**Authors:** Xin-hui Du, Hua Wei, Po Li, Wei-Tao Yao

**Affiliations:** ^1^Department of Orthopedics, Henan Cancer Hospital/Affiliated Cancer Hospital of Zhengzhou University, Zhengzhou, China; ^2^Department of Anesthesiology, The First Affiliated Hospital of Zhengzhou University, Zhengzhou, China

**Keywords:** pelvic osteosarcoma, artificial intelligence, image fusion, tumor margin, irregular bone

## Abstract

Surgeries of pelvic bone tumors are very challenging due to the complexity of anatomical structures and the irregular bone shape. CT and MRI are used in clinic for tumor evaluation, each with its own advantages and shortcomings. Combining the data of both CT and MRI images would take advantage of the merits of both images and provide better model for preoperative evaluation. We utilized an artificial intelligence (AI)-assisted CT/MRI image fusion technique and built a personalized 3-D model for preoperative tumor margin assessment. A young female patient with pelvic osteosarcoma was evaluated with our novel image fusion 3-D model in comparison with the 3-D model based solely on CT images. The fusion image model showed more detailed anatomical information and discovered multiple emboli within veins which were previously neglected. The discovery of emboli implied abysmal prognosis and discouraged any attempts for complex reconstruction after tumor resection. Based on the experience with this pelvic osteosarcoma, we believe that our image fusion model can be very informative with bone tumors. Though further validation with a large number of clinical cases is required, we propose that our model has the potential to benefit the clinic in the preoperative evaluation of bone tumors.

## Background

Based on the shapes, bones can be generally divided into long bones, short bones, flat bones, sesamoid bones, and irregular bones. Irregular bones are those that do not have any easily characterized shape, such as the spine, sacrum, and pelvic bones. Surgery of malignant bone tumors, either primary or metastatic, can be very challenging with irregular bones. Despite that systematic treatment options of cancer have been evolving quickly in recent years, surgery of bone malignancies still plays a very critical and non-replaceable role in the foreseeable future. Spines are the most common sites for cancer bone metastasis. Localized or radiating pain caused by bone destruction or impending paralysis caused by tumor invading the spinal cord is an indication for immediate surgeries (1). The sacrum is one of the most common sites for chordoma and giant cell tumor which requires surgical resection (2). Pelvic bones are common sites for chondrosarcoma and giant cell tumor and less commonly malignant tumors such as osteosarcoma and Ewing's sarcoma, in which cases surgeries are mandatory (3). Tumors arising in other irregular bones such as scapular can also be observed in clinic every now and then, forcing us to deal with these uncommon obstacles.

Most of the difficulties lie in the bizarre shapes of the bones and the complexity of adjacent structures such as blood vessels and nerves, daunting any attempt to properly assess tumor margins. Tumor margins directly determine surgical plans. Thus, proper delineation of tumor margins and evaluation of their relationship with adjacent structures are critical in preoperative planning.

Multiple attempts have been proposed to evaluate tumor margins. CT and MRI imaging are most commonly used in clinic to assess bone tumor margins, each with its own advantages and shortcomings. CT scan can show detailed information regarding bony destructions and calcifications but has limitations with soft tissue mass margin determination. MRI, on the other hand, can present sharp margins of soft tissue masses but could not distinguish changes within bones or calcification lesions properly. Despite their limitations, they do provide valuable information in tumor planning. MRI, for example, is believed to provide valuable information in osteosarcoma margin determination in extremities. In one study, the authors reviewed studies using MRI for extremity osteosarcoma margin evaluation and found that negative surgical margins were achieved in about 88.8–100% of the cases (4). Besides, CT images especially CT-guided surgery have long been used in pelvic tumor resection. Recently, multiple studies have shown that CT-guided surgery can help with better bony surgical margins in pelvic and sacral bones but have limitations with soft tissue margins (5, 6). Though each imaging data has been shown to be able to guide surgical plans in tumors of long bones, their individual contribution to irregular bone malignancy is still far from satisfactory.

Based on the feature of these imaging methods, it is reasonable to estimate that fusion of these two data can combine the merits of two methods and get more useful information to guide clinical decisions. Image fusion attempts have been initialized by combining images from different instruments such as ultrasound, CT, and MRI with multiple methods. Better results were reported compared using each of the image of individuals with kidney and prostate cancers (7, 8). However, no report is available by now regarding the role of fusion imaging in guiding bone tumor evaluation, especially those of irregular bones.

In this study, we for the first time utilized a novel 3-D model reconstructed with AI-assisted CT/MRI image fusion technique by combining enhanced CT and MRI imaging data to assess the tumor margin of a pelvic osteosarcoma. Our data showed that the image fusion model presented better demonstration of details of tumor margins and vascular emboli than any one of these image models or the 3-D model based on CT data alone.

## Case Presentation

**Figure 1:**
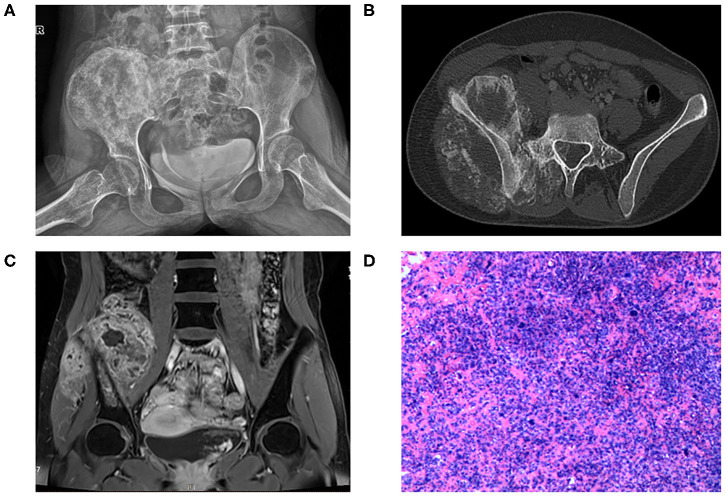
**(A)** X-ray of pelvic bone showing bone destruction and irregular calcification of the right ilium extending to the sacrum. **(B)** CT scan showing large soft tissue mass surrounding the right ilium with sunburst calcification. The tumor invades to L5 vertebral cavity. **(C)** T1 sequence with contrast of MRI showing huge soft tissue mass surrounding the ilium with mixed signals. **(D)** HE staining of the specimen showing high-grade anaplastic tumor with immature osteoid production.

A female patient 13 years of age was referred to our hospital due to increasing pain of the right hip for three months. She complained of body weight loss of about 5 kg and gait change. Physical examination showed swell and tenderness of the right hip. No significant alteration was detected in laboratory tests. X-ray (Figure 1A) and CT (Figure 1B) showed bone destruction and irregular calcification of the right ilium. MRI (Figure 1C) showed mixed signal in the right ilium and the huge surrounding soft tissue mass. She was a student at school by the time the disease occurred. The patient reported no history of cancer in her or her family.

**Figure 2:**
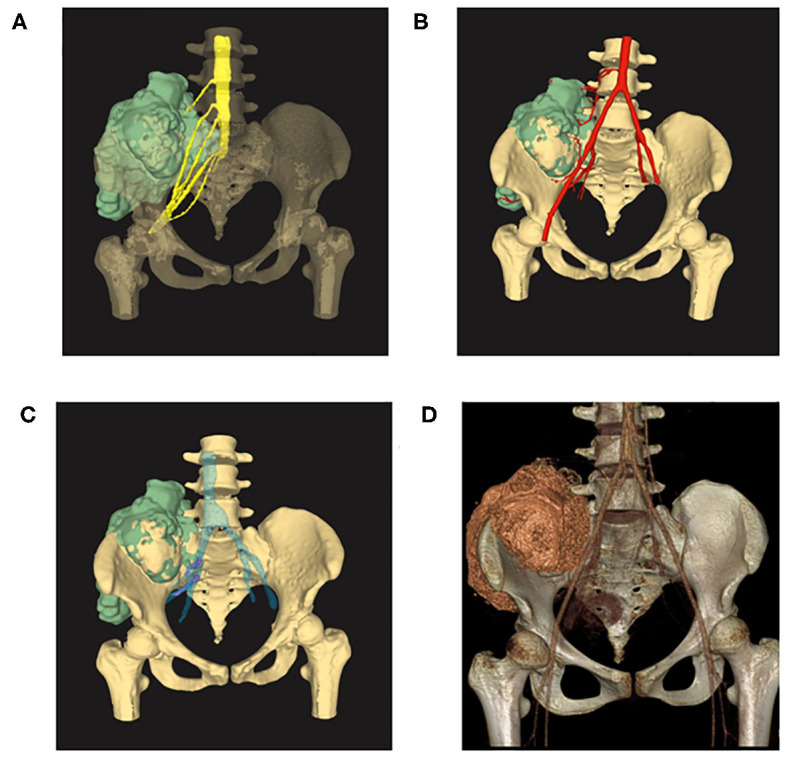
**(A)** 3-D model reconstructed with AI-assisted CT/MRI image fusion technique. Note that the tumor size was bigger and margins are better shown in this model comparing with the one with solely CT image data. **(B)** Image fusion data model showing tumor together with adjacent arteries. **(C)** Image fusion model showing tumor together with adjacent veins. Note that emboli were shown in purple within the veins. **(D)** 3-D model reconstructed with enhanced CT image data showing tumor of the right ilium extending to the sacrum. Note that the tumor did not invade L5 vertebra in this model.

Osteosarcoma was suspected and later confirmed with pathology after core needle biopsy (Figure 1D). Two cycles of neoadjuvant chemotherapy of methotrexate 10 g/m^2^, doxorubicin 60 mg/m^2^, and cisplatin 100 mg/m^2^ were planned preoperatively but disrupted after one cycle due to poor tolerance of the patient toward chemotherapy. The patient refused to continue with the chemotherapy and insisted to remove the tumor first. Tumor margin was reevaluated with enhanced CT and MRI imaging of the lesion after chemotherapy. AI-assisted CT/MRI image fusion technique was utilized, and a personalized 3-D pelvic model (Figures 2A–C) showing tumor was constructed together with adjacent structures such as blood vessels, nerves, and L5 vertebra. This fusion technique used was based on symmetric diffeomorphic image registration with cross-correlation (9–11). Each pixel of MRI information was matched to its corresponding CT images. The detailed technique was confidential. A normal 3-D pelvic model (Figure 2D) based solely on enhanced CT image data was also constructed as a control. We compared the tumor margins based on different models.

**Figure 3:**
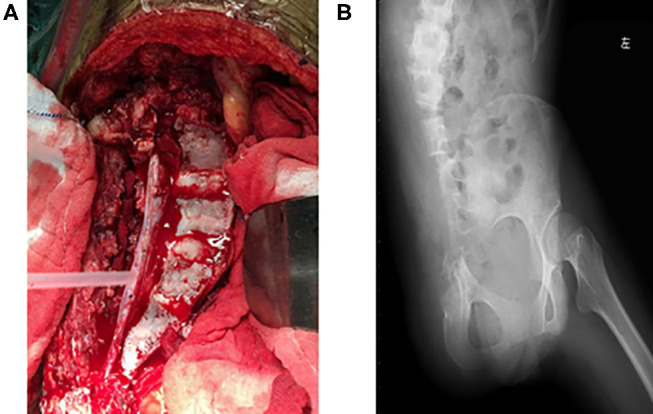
**(A)** Gross view of transverse resection of the sacrum and L5 during surgery. **(B)** Postoperative X-ray of the pelvis showing resection of the right ilium and semi-resection of the sacrum and L5 vertebra.

The image fusion model showed more precise margins especially with soft tissue extension while the CT alone-based model seemed to underestimate tumor size and lost much detail in margin delineation. Of note, emboli within the blood vessel which were initially neglected by CT or MRI alone model were clearly shown in our CT/MRI image fusion model. The discovery of emboli within the blood vessel which suggested metastasis discouraged any reconstruction surgery and promoted us to choose pelvic amputation in an attempt to get better local disease control (Figures 3A,B). Negative surgical margin was achieved, and the patient resumed adjuvant chemotherapy 3 weeks after surgery. The patient recovered well from the surgery and continued with adjuvant chemotherapy by adding IFO 10 g/m^2^ to the original chemotherapy plan. To our disappointment, lung metastasis was detected in the third cycle of adjuvant chemotherapy and the patient discontinued therapy. The patient died after 1 year of surgery due to lung metastasis but with no sign of local recurrence.

## Discussion

Osteosarcoma is a highly deadly disease mostly affecting skeletal immature adolescents (12). It preferentially occurs in long bones around the knee joint but can also present in other irregular bones such as pelvic bone in our case here. Pelvic osteosarcomas often are diagnosed later and with bigger volume than the ones occurring in long bones due to the fact that they are deeply located. To make it worse, pelvic osteosarcomas did not seem to respond well to neoadjuvant chemotherapy and the survival was more dismal than the ones in long bones (13, 14). In fact, Guo et al. suggested in one study that neo-chemotherapy does not seem to improve overall survival in pelvic osteosarcoma (15), which supported our decision to agree to discontinue neoadjuvant chemotherapy when the patient showed poor tolerance. Postoperative specimen was assessed by a pathologist, and the necrosis rate was <90%, which indicated poor response to neoadjuvant chemotherapy.

With the depressing performance of systematic therapy in pelvic sarcoma, it seems that the only available option for now is to improve our surgery to get better local control. However, pelvic tumor surgeries are always challenging and with high complication rates (16, 17). Thus, proper preoperative planning is of vital importance.

## Significance

We for the first time utilized the AI-assisted CT/MRI fusion model to guide pelvic sarcoma preoperative evaluation. Each pixel of MRI information was matched to its corresponding CT images based on symmetric diffeomorphic image registration with cross-correlation. As mentioned previously, CT scan could show good bony margins but could not show detailed information of the soft tissue mass. However, MRI could provide more accurate information on soft tissue mass but did poorly with bone destructions. Our model combines the merits of the two techniques and could provide more detailed information to precisely show the tumor and benefit clinical evaluation.

This technique can benefit clinic beyond pelvic tumors. In fact, it is most advantageous in tumors occurring in irregular bones such as vertebra, pelvic bones, scapula, and bones of hand and feet. In addition to guiding tumor margin evaluation, personalized instruments such as guide plate or implant could also be developed based on our model, which could further help us in clinic.

## Data Availability Statement

All datasets generated for this study are included in the article/supplementary material.

## Ethics Statement

Written informed consent was obtained from the patient's legal representative for the publication of this study.

## Author Contributions

XD: paper writing and review. W-TY: figure preparation. PL: advised on the paper draft. HW: literature review. All authors contributed to the article and approved the submitted version.

## Conflict of Interest

The authors declare that the research was conducted in the absence of any commercial or financial relationships that could be construed as a potential conflict of interest.
